# Supplementary material for: The Mobilome-Enriched Genome of the Competence-Deficient Streptococcus pneumoniae BM6001, the Original Host of Integrative Conjugative Element Tn5253, Is Phylogenetically Distinct from Historical Pneumococcal Genomes
Source: Microorganisms. 2023 Jun 23;11(7):1646. doi: 10.3390/microorganisms11071646 (PMC10383233; doi:10.3390/microorganisms11071646)
Supplement: Supplementary file 1 [file microorganisms-11-01646-s001.zip › Table S1 Colombini et al.pdf]

Table S1. Oligonucleotide primers.

Name	Sequence (5' to 3')	Target	Amplified Product (bp)	Position on BM6001 Genome (CP107038)
IF1255	GAACCACTAGTCACATAGGAT	ΦBM6001.2, left chromosomal junction	ΦBM6001.2 reconstituted target site (267)	1,556,538 – 1,556,558
IF1258	CTAATATCGCTCGGACAGTTT	ΦBM6001.2, right chromosomal junction		1,599,227 – 1,599,247
IF1256	ACGGAACACTGGCAGACAA	ΦBM6001.2, 5' end	ΦBM6001.2 episomal form (424)	1,556,832 – 1,556,850
IF1257	CTACTGAAGATATGGAGGAT	ΦBM6001.2, 3' end		1,598,872 – 1,598,891
IF1259	TCGGGTATACTATAGACGGTT	ΦBM6001.1, left chromosomal junction	ΦBM6001.1 reconstituted target site (239)	41,958 – 41,978
IF1262	GCAATCTCAGCCTCTCTCAA	ΦBM6001.1, right chromosomal junction		78,283 – 78,302
IF1260	CGAACTTGCTGAAGCGACA	ΦBM6001.1, 5' end	ΦBM6001.1 episomal form (479)	42,223 – 42,241
IF1261	CGGTCACTAGACTTGCGAT	ΦBM6001.1, 3' end		77,870 – 77,888
IF1263	CGCCCATCTGCTTGTAACTT	ΦBM6001.3, left chromosomal junction	ΦBM6001.3 reconstituted target site (373)	2,042,119 – 2,042,138
IF1266	GAGTCAACCGCACCATGAA	ΦBM6001.3, right chromosomal junction		2,081,334 – 2,081,352
IF1264	GGATAGAGGACCAAGAGGTT	ΦBM6001.3, 5' end	ΦBM6001.3 episomal form (332)	2,042,410 – 2,042,429
IF1265	TACAGAGCAAGTCAGCCGAA	ΦBM6001.3, 3' end		2,080,923 – 2,080,942
IF1267	AGCAGATACTGACGAAGACTT	ΦBM6001.4, left chromosomal junction	ΦBM6001.4 reconstituted target site (700)	2,603 – 2,623
IF1271	CAATCGCATCTACTTCACGAA	ΦBM6001.4, right chromosomal junction		21,866 – 21,886
IF1268	TACCTATCAGCAACCAGCAA	ΦBM6001.4, 3' end	ΦBM6001.4 episomal form (361)	3,163 – 3,182

Name	Sequence (5’ to 3’)	Target	Amplified Product (bp)	Position on BM6001 Genome (CP107038)
IF1270	TAGCCACCTATCCAAAGACAA	ΦBM6001.4, 5’ end		21,388 – 21,408
IF1272	AGTCGGATTGACGAGATTGAT	ΦBM6001.5, left chromosomal junction	ΦBM6001.5 reconstituted target site (338)	228,659 – 228,679
IF1276	GACTTCCCTGAACCTGACAA	ΦBM6001.5, right chromosomal junction		241,879 – 241,860
IF1273	TAAGCGTGCTGATGTTCCTA	ΦBM6001.5, 5’ end	ΦBM6001.5 episomal form (496)	229,119- 229,100
IF1275	TGCCTGCTGAACTCGTCAA	ΦBM6001.5, 3’ end		241,509 – 241,527
IF1378	GTTTTGATGACCGATGCCGAT	Chromosomal *gyrB* gene, 5’ end	Chromosomal reference gene (144)	808,406 – 808,426
IF1379	GCTTCCAACCTTGACACCAT	Chromosomal *gyrB* gene, 3’ end		808,549 – 808,530
IF1543	CTTCGTCTTGTCATCAACCAA	Tn*7089*, left chromosomal junction	Tn*7089* reconstituted target site (416)	339,149 – 339,169
IF1544	AAGTCGCAACAACTGTGTCAA	Tn*7089*, right chromosomal junction		348,600 – 348,580
IF1545	CAGAAAGTCTGAATAAGTGTAAAT	Tn*7089*, 3’ end	Tn*7089* circular form (525)	348,075 – 348,098
IF1546	TCTGCTGGACAAGTTGCCTA	Tn*7089*, 5’ end		339,542 – 339,561
IF1579	TCTGGCGTCATATTCGTAACT	Tn*7090*, left chromosomal junction	Tn*7090* reconstituted target site (773)	1,920,213 – 1,920,233
IF1580	TTTGCTGAGGCTAGGGTGTT	Tn*7090*, right chromosomal junction		1,930,038 – 1,930,019
IF1581	GCAAGAGTTAATAGAAAGTGTGA	Tn*7090*, 3’ end	Tn*7090* circular form (807)	1,929,203 – 1,929,225
IF1588	TGTGTTGAATCCAGAGTAGTA	Tn*7090*, 5’ end		1,920,938 – 1,920,958
