# Supplementary material for: The Mobilome-Enriched Genome of the Competence-Deficient Streptococcus pneumoniae BM6001, the Original Host of Integrative Conjugative Element Tn5253, Is Phylogenetically Distinct from Historical Pneumococcal Genomes
Source: Microorganisms. 2023 Jun 23;11(7):1646. doi: 10.3390/microorganisms11071646 (PMC10383233; doi:10.3390/microorganisms11071646)
Supplement: Supplementary file 1 [file microorganisms-11-01646-s001.zip › Table S2 Colombini et al.pdf]

Table S2. Insertion Sequences in the *S. pneumoniae* BM6001 genome.

**IS Family**	**IS Name** [a]	**Copies** [b]
IS*1380*	IS*Spn5*	17 (2)
IS*L3*	IS*1167*	16 (12)
IS*5* sub-group IS*L2*	IS*Spn7*	11 (10)
	IS*1381A*	1 (1)
IS*630*	IS*630-Spn1*	5 (5)
	IS*Spn2*	4 (4)
IS*3* sub-group IS*150*	IS*Spn11*	6 (5)
IS*110*	IS*Bth13*	3 (2)
IS*30*	IS*Spn8*	3 (1)
IS*66*	-	3 (3)
IS*1182*	IS*Smi2*	2 (1)
IS*200*/IS*605* sub-group IS*200*	IS*200S*	1 (1)
IS*5* sub-group IS*1031*	IS*Wpi1*	1

[a] For family IS*66* members, IS name was not identified due to the presence of a truncated transposase gene. [b] The number reported in parenthesis refers to the copies of ISs containing a truncated or frame-shifted transposase gene.
