# Supplementary material for: The Mobilome-Enriched Genome of the Competence-Deficient Streptococcus pneumoniae BM6001, the Original Host of Integrative Conjugative Element Tn5253, Is Phylogenetically Distinct from Historical Pneumococcal Genomes
Source: Microorganisms. 2023 Jun 23;11(7):1646. doi: 10.3390/microorganisms11071646 (PMC10383233; doi:10.3390/microorganisms11071646)
Supplement: Supplementary file 1 [file microorganisms-11-01646-s001.zip › Table S3 Colombini et al.pdf]

Table S3. Annotated ORFs of BM6001 prophages and satellite prophages.

Prophage	ORF (aa) [a]	Predicted Protein	Virfam Homologous Protein (Identity) [E Value/Probability]	Homologous Protein ID/Origin Identity (%) [E Value] [b]	Pfam Domain [c] (aa) [E Value]
ΦBM6001.1	*orf1* (382)	Tyrosine-type DNA integrase		APD23317.1/ *Streptococcus* phage IPP42 382/382 (100%) [0.0]	Integrase catalytic domain (192-367) [9.0e-16]
	orf2 (266)	HIRAN domain-containing protein		APD23318.1/ *Streptococcus* phage IPP42 256/256 (100%) [0.0]	HIRAN domain (175-233) [2.60e-08]
	orf3 (251)	CI-like repressor		APD23319.1/ *Streptococcus* phage IPP42 251/251 (100%) [0.0]	Cro/C1-type helix-turn-helix domain (13-72) [1.20e-06]; Peptidase S24/S26A/S26B/S26C (132-243) [1.40e-22]
	orf4 (68)	CI-like repressor		APD23320.1/ *Streptococcus* phage IPP42 68/68 (100%) [2e-51]	Cro/C1-type helix-turn-helix domain (6-60) [2.8e-12]
	orf6 (237)	Antirepressor protein		APD23323.1/ *Streptococcus* phage IPP42 237/237 (100%) [0.0]	BRO-N-domain (1-104) [24.244535]; ORF6C-domain (115-228) [1.0e-35]
	orf8 (106)	Putative lipoprotein		APD23325.1/ *Streptococcus* phage IPP42 106/106 (100%) [1e-78]	
	orf9 (97)	Replication initiation protein		APD23326.1/ *Streptococcus* phage IPP42 97/97 (100%) [2e-61]	
	orf10 (275)	Replication initiation protein		APD23327.1/ *Streptococcus* phage IPP42 275/275 (100%) [0.0]	Phage replisome organiser, N-terminal domain or DnaD domain (6-125) [7.5e-46]; Replication initiation and membrane attachment (186-246) [3.3e-10]
	orf11 (256)	DNA replication protein		APD23329.1/ *Streptococcus* phage IPP42 256/256 (100%) [2e-162]	IstB-like ATP-binding protein (56-118) [2.4-12]
	orf19 (117)	CI-like repressor		APD23336.1/ *Streptococcus* phage IPP42 117/117 (100%) [6e-70]	Cro/C1-type helix-turn-helix domain (5-58) [5.2e-11]
	orf20 (154)	Transcriptional activator		APD23337.1/ *Streptococcus* phage IPP42 153/154 (99%) [1e-107]	
	orf21 (180)	Tyrosine-type DNA integrase		APD23338.1/ *Streptococcus* phage IPP42 180/180 (100%) [3e-127]	Integrase catalytic domain (2-176) [26.76766]
	orf22 (68)	HNH endonuclease		APD23339.1/ *Streptococcus* phage IPP42 68/68 (100%) [4e-45]	HNH endonuclease (1-51) [1.61182e-04]

Prophage	ORF (aa) [a]	Predicted Protein	Virfam Homologous Protein (Identity) [E Value/Probability]	Homologous Protein ID/Origin Identity (%) [E Value] [b]	Pfam Domain [c] (aa) [E Value]
	orf23 (161)	TPA: repressor		APD23340.1/ *Streptococcus* phage IPP42 158/159 (99%) [4e-107]	
	orf24 (570)	Terminase large subunit	TerminaseL (29%) [6e-53]	APD23341.1/ *Streptococcus* phage IPP42 570/570 (100%) [0.0]	Terminase large subunit endonuclease domain (278-555) [2.4e-32]; Terminase large subunit ATPase domain (107-265) [6.4e-19]
	orf25 (380)	Phage portal protein	Portal (19%) [100%]	APD23342.1/ *Streptococcus* phage IPP42 380/380 (100%) [0.0]	Bacteriophage/Gene transfer agent portal protein (47-351) [2.8e-21]
	orf26 (180)	HK97 family phage prohead protease	Major capsid protein (28%) [100%]	APD23343.1/ *Streptococcus* phage IPP42 180/180 (100%) [3e-128]	Caudovirus prohead serine protease (8-152) [1.7e-39]
	orf27 (417)	Phage major capsid protein		APD23344.1/ *Streptococcus* phage IPP42 417/417 (100%) [0.0]	Phage major capsid gp5 (132-415) [4.45e-33]
	orf28 (111)	Phage head-tail adapter protein	Adaptor (18%) [94,63%]	APD23345.1/ *Streptococcus* phage IPP42 111/111 (100%) [3e-76]	
	orf29 (101)	Phage head-tail adapter protein	Head-closure (18%) [94.01%]	APD23346.1/ *Streptococcus* phage IPP42 101/101 (100%) [2e-67]	Phage head-tail joining protein (20-91) [3.8e-05]
	orf30 (115)	Ras-related GTPase protein	Tail completion (21%) [88.69%]	APD23347.1/ *Streptococcus* phage IPP42 115/115 (100%) [1e-81]	
	orf32 (222)	Phage tail protein	Major Tail Protein (22%) [1e-09]	APD23349.1/ *Streptococcus* phage IPP42 222/222 (100%) [6e-156]	Phage tail tube protein, N-terminal (3-196) [6.0e-41]
	orf34 (912)	Phage tail tape measure protein		APD23351.1/ *Streptococcus* phage IPP42 912/912 (100%) [0.0]	Phage related minor tail protein (300-469) [4.9e-13]
	orf35 (240)	Phage tail protein		APD23352.1/ *Streptococcus* phage IPP42 240/240 (100%) [2e-165]	
	orf36 (3,035)	Tail fiber domain-containing protein		APD23353.1/ *Streptococcus* phage IPP42 3016/3035 (99.37%) [0.0]	Phage minor structural protein, N-terminal domain (28-321) [4.2e-31]; Intramolecular chaperone auto-processing domain (2941-2992) [3.3e-12]
	orf40 (138)	Phage holin family		APD23357.1/ *Streptococcus* phage IPP42	Bacteriophage holin family (22-126) [1.62-

Prophage	ORF (aa) [a]	Predicted Protein	Virfam Homologous Protein (Identity) [E Value/Probability]	Homologous Protein ID/Origin Identity (%) [E Value] [b]	Pfam Domain [c] (aa) [E Value]
		protein		138/138 (100%) [3e-94]	12]
	orf41 (110)	Phage holin family protein		APD23358.1/ *Streptococcus* phage IPP42 110/110 (100%) [8e-4]	Phage holin LL-H family (5-105) [1.1e-27]
	orf42 (318)	N-acetylmuramoyl-L-alanine amidase LytA		APD23359.1/ *Streptococcus* phage IPP42 318/318 (100%) [0.0]	N-acetylmuramoyl-L-alanine amidase domain (19-151) [3.0-06]
ΦBM6001.2	*orf1* (318)	N-acetylmuramoyl-L-alanine amidase LytA		ALA47323.1/ *Streptococcus* phage phiARI0131-1 310/318 (97.48%) [0.0]	N-acetylmuramoyl-L-alanine amidase domain (19-151) [.2e-06]
	orf2 (109)	Phage holin family protein		ALA47324.1/ *Streptococcus* phage phiARI0131-1 102/109 (95%) [2e-68]	Bacteriophage holin of superfamily 6; Holin_LLH (5-105) [4.1e-27]
	orf6 (2,386)	Tail fiber domain-containing protein		ALA47326.1/ *Streptococcus* phage phiARI0131-1 1247/1658 (75%) [0.0]	Intramolecular chaperone auto-processing domain (2292-2342) [3.8e-12]
	orf11 (137)	Phage tail protein	Major Tail Protein (24%) [4e-10]	ALA47330.1/ *Streptococcus* phage phiARI0131-1 137/137 (100%) [2e-91]	
	orf12 (122)	Phage tail protein	Tail completion (30%) [100%]	ALA47331.1/ *Streptococcus* phage phiARI0131-1 122/122 (100%) [9e-86]	
	orf13 (171)	HK97 gp10 family phage protein	Ne1 (28%) [100%]	YP_009320687.1/ *Streptococcus* phage phiARI0131-1 171/171 (99.42%) [1e-131]	
	orf14 (112)	Phage head-tail adapter protein	Head-closure (23%) [100%]		
	orf15 (103)	Phage head-tail adapter protein	Adaptor protein (27%) [100%]	AHN84635.1/ *Streptococcus* phage Spn1 102/103 (90.03%) [3e-64]	
	orf17 (60)	Rho termination factor N-terminal domain-containing protein		ALA47334.1/ *Streptococcus* phage phiARI0131-1 60/60 (100%) [9e-39]	Rho termination factor, N-terminal domain superfamily (18-53) [1.06e-05]
	orf18 (281)	N4-gp56 family major capsid protein	Major capsid protein (50%) [100%]	ALA47335.1/ *Streptococcus* phage phiARI0131-1 280/281 (99%) [0.0]	Major capsid protein, N4-gp56 family (5-276) [3.9e-29]
	orf22 (75)	16S rRNA processing protein RimM		APD21949.1/ *Streptococcus* phage IPP15 75/75 (100%) [1e-46]	
	orf23 (467)	Phage minor capsid		ALA47341.1/ *Streptococcus* phage	Phage Mu F like protein (198-303) [1.6e-

Prophage	ORF (aa) [a]	Predicted Protein	Virfam Homologous Protein (Identity) [E Value/Probability]	Homologous Protein ID/Origin Identity (%) [E Value] [b]	Pfam Domain [c] (aa) [E Value]
		protein		phiARI0131-1 322/333 (97%) [0.0]	14]
	orf24 (489)	Phage portal protein	Portal (29%) [100%]	ALA47342.1/ *Streptococcus* phage phiARI0131-1 486/489 (99.39%) [0.0]	Phage portal protein SPP1 Gp6-like (24-441) [1.9e-99]
	orf25 (432)	PBSX family phage terminase large subunit	TermL (60%) [1e-155]	ALA47343.1/ *Streptococcus* phage phiARI0131-1 425/432 (99%) [0.0]	Phage terminase large subunit N-terminal (32-238) [8.9e-73]; Phage terminase large subunit C-terminal (271-415) [5.7e-27]
	orf26 (146)	Terminase small subunit		ALA47344.1/ *Streptococcus* phage phiARI0131-1 146/146 (100%) [2e-99]	Terminase small subunit (7-131) [6.5e-31]
	orf27	tRNA-tyr			
	orf31 (131)	YopX family protein			YopX protein (4-127) [2.2e-16]
	orf38 (449)	Virulence-associated E family protein		ALA47351.1/ *Streptococcus* phage phiARI0131-1 448/449 (99%) [0.0]	Virulence-associated protein E (98-363) [3.6e-48]
	orf39 (273)	Bifunctional DNA primase/polymerase		ALA47352.1/ *Streptococcus* phage phiARI0131-1 273/273 (100%) [0.0]	Bifuntional DNA primase/polymerase N-terminal (8-148) [6.6e-21]<br>Primase C-terminal 1 (214-270) [2.1e-10]
	orf41 (275)	ATP-binding protein		ALA47354.1/ *Streptococcus* phage phiARI0131-1 275/275 (100%) [0.0]	AAA domain (18-197) [1.2e-24]
	orf43 (397)	Type III restriction endonuclease subunit R		ALA47356.1/ *Streptococcus* phage phiARI0131-1 396/397 (99%) [0.0]	Helicase superfamily 1/2, ATP-binding domain (1-152) [10.087828]
	orf46 (160)	Siphovirus Gp157 family protein		ALA47359.1/ *Streptococcus* phage phiARI0131-1 160/160 (100%) [4e-107]	Siphovirus Gp157 (4-160) [1.9e-38]
	orf47 (254)	HNH endonuclease		ALA47360.1/ *Streptococcus* phage phiARI0131-1 240/240 (100%) [4e-169]	HNH endonuclease (176-217) [3.4e-06]
	orf58 (71)	CI-like repressor		ALA47317.1/ *Streptococcus* phage phiARI0131-1 70/71 (99%) [4e-48]	Cro/C1-type helix-turn-helix domain (5-59) [2.7e-10]
	orf61 (263)	CI-like repressor		ALA47320.1/ *Streptococcus* phage phiARI0131-1 263/263 (100%) [0.0]	Cro/C1-type helix-turn-helix domain (14-68) [13.48797]; Peptidase S24/S26A/S26B/S26C (159-255) [5.4e-19]
	orf62 (316)	Abortive infection		ALA47321.1/ *Streptococcus* phage	Abi-like protein (30-229) [7.6e-39]

Prophage	ORF (aa) [a]	Predicted Protein	Virfam Homologous Protein (Identity) [E Value/Probability]	Homologous Protein ID/Origin Identity (%) [E Value] [b]	Pfam Domain [c] (aa) [E Value]
		system protein AbiD/AbiF-like		phiARI0131-1 315/316 (99%) [0.0]	
	orf63 (373)	Tyrosine-type DNA integrase		ALA47322.1/ *Streptococcus* phage phiARI0131-1 373/373 (100%) [0.0]	Integrase catalytic domain (174-358) [7.2e-29]
ΦBM6001.3	*orf2* (318)	N-acetylmuramoyl-L-alanine amidase family protein		CAC48116.1/ *Streptococcus* phage MM1 308/318 (97%) [0.0]	N-acetylmuramoyl-L-alanine amidase domain (10-151) [1.1e-26]
	orf3 (110)	Phage holin family protein		APD23626.1/ *Streptococcus* phage IPP48 110/110 (98.18%) [5e-48]	Bacteriophage holin of superfamily 6 (5-105) [6.3e-27]
	orf4 (138)	Phage holin family protein		CAC48114.1/ *Streptococcus* phage MM1 138/138 (100%) [3e-92]	Bacteriophage holin family (21-126) [7.5e-13]
	orf7 (2,005)	Phage tail protein		CAC48111.1/ *Streptococcus* phage MM1 1799/2018 (89%) [0.0]	Galactose binding like domain (1691-1819) [5.31e-5]
	orf8 (504)	Phage tail protein		CAC48105.1/ *Streptococcus* phage MM1 503/504 (99.80%) [0.0]	Siphovirus-type tail component (18-504) [6.7-44]
	orf9 (1,093)	Phage tape measure protein		CAC48109.1/ *Streptococcus* phage MM1 1053/1093 (96%) [0.0]	Tape measure protein N-terminal (64-255) [1.5e-37]
	orf10 (189)	Bacteriophage GP15 family protein		CAC48108.1/ *Streptococcus* phage MM1 188/188 (100%) [3e-142]	Bacteriophage A500 Gp15 protein (5-181) [1.4e-39]
	orf11 (162)	Phage tail assembly chaperonine		CAC48107.1/ *Streptococcus* phage MM1 161/162 (99%) [7e-122]	
	orf12 (149)	Phage major tail shaft protein	Major tail protein (97%) [5e-83]	CAC48106.1/ *Streptococcus* phage MM1 145/149 (97.32%) [2e-90]	
	orf13 (135)	Phage minor capsid protein	Tail completion (99%) [100%]	CAC48105.1/ *Streptococcus* phage MM1 134/135 (99%) [9e-90]	Bacteriophage minor capsid protein (24-129) [1.9e-13]
	orf14 (114)	Phage minor capsid protein	Ne1 protein (98%) [100%]	CAC48104.1/ *Streptococcus* phage MM1 112/114 (98%) [2e-85]	Minor capsid protein (4-112) [2.5e-31]
	orf15 (123)	Phage minor capsid protein	Head-closure (99%) [100%]	CAC48103.1/ *Streptococcus* phage MM1 122/123 (99%) [7e-79]	Minor capsid protein (12-122) [1.3e-16]

**Prophage**	**ORF (aa)** [a]	**Predicted Protein**	**Virfam Homologous Protein (Identity) [E Value/Probability]**	**Homologous Protein ID/Origin Identity (%) [E Value]** [b]	**Pfam Domain** [c] **(aa) [E Value]**
	orf16 (130)	Phage head-tail adapter protein	Adaptor (99%) [100%]	CAC48102.1/ *Streptococcus* phage MM1 129/130 (99%) [2e-83]	
	orf17 (77)	Phage major capsid protein	Major capsid protein (95%) [100%]	CAC48101.1/ *Streptococcus* phage MM1 77/77 (100%) [2e-34]	
	orf18 (293)	Phage major capsid protein		APD21898.1/ *Streptococcus* phage IPP14 293/293 (100%) [0.0]	
	orf19 (187)	Phage scaffolding protein		CAC48099.1/ *Streptococcus* phage MM1 187/187 (100%) [1e-117]	Capsid assembly scaffolding protein (16-160) [3.9e-31]
	orf20 (383)	Phage minor capsid protein		CAC48098.1/ *Streptococcus* phage MM1 381/383 (99%) [0.0]	Structural protein from Lactococcus phage (11-365) [4.0e-88]
	orf22 (522)	Phage portal protein	Portal protein (99%) [100%]	YP_009322103.1/ *Streptococcus* phage phiARI0468-4 518/522 (99.42%) [0.0]	Portal protein A118-type (1-514) [6.2e-259]
	orf23 (436)	Terminase large subunit	TermL (99%) [0]	CAC48095.1/ *Streptococcus* phage MM1 432/436 (99%) [0.0]	Bacteriophage Terminase large subunit (24-425) [6.8e-79]
	orf24 (151)	Stress-induced protein		CAD29474.1/ *Streptococcus* phage MM1 149/151 (98.68%) [3e-101]	Stress-induced bacterial acidophilic repeat motif (15-32) [2.4e-4]
	orf26 (364)	Chromosome partitioning protein parB		CAD29472.1/ *Streptococcus* phage MM1 362/364 (99%) [0.0]	ParB/Sulfredoxin domain (6-144) [3.66e-24]
	orf32 (133)	Endodeoxyribonuclease RusA-like protein		CAC48085.1/ *Streptococcus* phage MM1 362/364 (99%) [0.0]	Holliday junction resolvase RusA-like superfamily (1-133) [3.92e-24]
	orf34 (179)	Single-stranded DNA-binding protein		CAC48078.1/ *Streptococcus* phage MM1 167/180 (93%) [4e-125]	Primosome PriB/single-strand DNA-binding (2-103) [2.4e-34]
	orf37 (233)	Essential recombination function (ERF) family protein		CAC48076.1/ *Streptococcus* phage MM1 231/235 (98%) [2e-132]	Essential recombination function protein (6-158) [4.5e-22]
	orf43 (117)	Helix-turn-helix domain-containing protein		APD24585.1/ *Streptococcus* phage IPP69 103/117 (88%) [2e-79]	
	orf45 (238)	Phage anti-repressor KilAC domain-		APD24583.1/ *Streptococcus* phage IPP69 238/238 (100%) [0.0]	AntA/AntB anti-repressor (17-82) [4.0e-25]; Anti-repressor protein, C-terminal

Prophage	ORF (aa) [a]	Predicted Protein	Virfam Homologous Protein (Identity) [E Value/Probability]	Homologous Protein ID/Origin Identity (%) [E Value] [b]	Pfam Domain [c] (aa) [E Value]
		containing protein			(121-231) [2.1e-34]
	orf46 (70)	Helix-turn-helix domain-containing protein		APD24582.1/ *Streptococcus* phage IPP69 70/70 (100%) [1e-51]	Lambda repressor-like DNA-binding domain (8-69) [8.26e-7]
	orf47 (243)	CI-like repressor		APD24581.1/ *Streptococcus* phage IPP69 242/243 (99%) [0.0]	Cro/C1-type helix-turn-helix domain (3-68) [2.4e-9]; Peptidase S24/S26C/S26B/S26C (126-238) [4.4e-24]
	orf49 (481)	Phage integrase		APD24579.1/ *Streptococcus* phage IPP69 468/481 (97%) [0.0]	Resolvase N-terminal catalytic domain (7-155) [1.3e-42]; DNA-binding recombinase domain (179-261) [3.0e-15]; Recombinase zinc beta ribbon domain (281-352) [2.7e-8]
ΦBM6001.4	*orf4* (113)	Replication protein		QBX10267.1 / *Streptococcus* satellite phage Javan428 113/113 (100%) [2e-74]	
	orf8 (484)	DNA primase		QBX13282.1/ *Streptococcus* satellite phage Javan759 484/484 (100%) [0.0]	D5 N terminal like (87-173) [1.3e-8]
	orf9 (286)	DNA primase		QBX13281.1/ *Streptococcus* satellite phage Javan759 286/286 (100%) [0.0]	Primase C1 terminal 1 (217-283) [5.3e-6]
	orf15 (46)	Glycerate kinase		QBX13275.1/ *Streptococcus* satellite phage Javan759 46/46 (100%) [2-23]	
	orf23 (201)	Rha family transcriptional regulator		QBX13266.1/ *Streptococcus* satellite phage Javan759 201/201 (100%) [4e-146]	Bacteriophage regulatory protein Rha family (14-99) [2.5e-13]
	orf25 (63)	Helix-turn-helix transcriptional regulator		QBX13264.1/ *Streptococcus* satellite phage Javan759 62/63(98% [8e-47]	Lambda repressor-like DNA-binding domain (5-61) [5.63e-15]
	orf26 (246)	CI-like repressor		QBX13263.1/ *Streptococcus* satellite phage Javan759 246/246 (100%) [3e-178]	Cro/C1-type helix-turn-helix domain (14-66) [2.5e-06]; Peptidase S24/S26A/S26B/S26C (130-240) [1.9e-19]
	orf27 (222)	Zinc ribbon domain-containing protein		QBX12573.1/ *Streptococcus* satellite phage Javan729 222/222 (100%) [2e-126]	
	orf28 (222)	Adenosine		QBX13261.1/ *Streptococcus* satellite phage	Fic/DOC family (74-173) [1.5e-20]

**Prophage**	**ORF (aa)** [a]	**Predicted Protein**	**Virfam Homologous Protein (Identity) [E Value/Probability]**	**Homologous Protein ID/Origin Identity (%) [E Value]** [b]	**Pfam Domain** [c] **(aa) [E Value]**
		monophosphate protein transferase		Javan759 222/222 (100%) [1e-163]	
	orf31 (101)	Replication protein		QBX13257.1/ *Streptococcus* satellite phage Javan759 101/101 (100%) [2e-66]	Replication protein (67-95) [6.1e-09]
	orf32 (186)	DNA damage inducible protein D, Bro family protein		QBX13256.1/ *Streptococcus* satellite phage Javan759 186/186 (100%) [3e-128]	BRO N-terminal domain (14-104) [2.3e-06]
	orf33 (388)	Tyrosine-type DNA integrase		QBX13330.1/ *Streptococcus* satellite phage Javan761 383/388 (100%) [2e-66]	Integrase SAM-like N-terminal domain (69-125) [8.6e-16]; Integrase catalytic domain (181-368) [1.0e-29]
ΦBM6001.5	*orf1* (388)	Tyrosine-type DNA integrase		QBX13210.1/ *Streptococcus* satellite phage Javan757 388/388 (100%) [0.0]	Integrase catalytic domain (186-371) [3.9e-27]; Integrase SAM-like N-terminal domain (71-125) [2.3e-16]
	orf2 (504)	Putative DNA-binding domain-containing protein		QBX13211.1/ *Streptococcus* satellite phage Javan757 504/504 (100%) [0.0]	Putative DNA-binding domain (14-137) [7.2e-14]; Putative ATP-dependent DNA helicase recG C-terminal (323-406) [2.0e-11]; BlaI transcriptional regulator (422-482) [5.1e-06]
	orf3 (237)	CI-like repressor		QBX13212.1/ *Streptococcus* satellite phage Javan757 237/237 (94.09%) [2e-146]	Cro/CI-type helix-turn-helix domain (22-71) [4.2e-09]
	orf5 (208)	Rha family transcriptional regulator		QBX13214.1/ *Streptococcus* satellite phage Javan757 208/208 (100%) [5e-144]	Bacteriophage regulatory protein Rha family (15-99) [2.7e-11]
	orf6 (150)	Glycerate kinase		QBX13215.1/ *Streptococcus* satellite phage Javan757 150/150 (100%) [7e-102]	
	orf7 (66)	Glycerate kinase		QBX13216.1/ *Streptococcus* satellite phage Javan757 66/66 (100%) [3e-42]	
	orf10 (93)	Transcriptional regulator		QBX13219.1/ *Streptococcus* satellite phage Javan757 93/93 (100%) [2e-61]	Putative DNA-binding domain (37-86) [6.04e-06]
	orf11 (288)	DNA primase		QBX13221.1/ *Streptococcus* satellite phage Javan757 288/288 (100%) [0.0]	Primase C-terminal domain (219-285) [5.4e-06]
	orf12 (492)	DNA primase		QBX13222.1/ *Streptococcus* satellite phage	DNA primase phage/plasmid (134-406)

**Prophage**	**ORF (aa)** [a]	**Predicted Protein**	**Virfam Homologous Protein (Identity) [E Value/Probability]**	**Homologous Protein ID/Origin Identity (%) [E Value]** [b]	**Pfam Domain** [c] **(aa) [E Value]**
				Javan757 491/492 (99.80%) [0.0]	[1.5e-64]

[a] The number of amino acids of the predicted protein is shown in parenthesis. [b] Determined by compositional matrix adjustment. [c] Numbers in parentheses represent the part of the protein homologous to the Pfam domain.
