# Supplementary material for: The Mobilome-Enriched Genome of the Competence-Deficient Streptococcus pneumoniae BM6001, the Original Host of Integrative Conjugative Element Tn5253, Is Phylogenetically Distinct from Historical Pneumococcal Genomes
Source: Microorganisms. 2023 Jun 23;11(7):1646. doi: 10.3390/microorganisms11071646 (PMC10383233; doi:10.3390/microorganisms11071646)
Supplement: Supplementary file 1 [file microorganisms-11-01646-s001.zip › Table S4 Colombini et al.pdf]

Table S4. Annotated ORFs of BM6001 genomic islands.

Genomic Island	ORF (aa) [a]	Predicted Protein	Homologous Protein ID/Origin Identity (%) [E Value] [b]	Pfam Domain [c] (aa) [E Value]
GI-BM6001.1	*orf2* (457)	ThiF family adenylyltransferase	WP_078064025.1/ *Streptococcus pneumoniae* TIGR4 262/265 (99%) [0.0]	THIF-type NAD/FAD binding fold (87-312) [4.6-38]
	orf3 (277)	ABC transporter ATP-binding protein, truncated	WP_000061739.1/ *Streptococcus pneumoniae* TIGR4 223/247(90%) [3e-98]	
	orf4 (182)	ABC transporter permease	WP_000919274.1/ *Streptococcus pneumoniae* TIGR4 177/182(97%) 2e-88]	ABC-2 family transporter protein (7-174) [6.7e-08]
	tnp (33)	IS*30* family trasposase, truncated		
GI-BM6001.2	*orf2* (341)	Periplasmic binding protein	WP_000790743.1/ *Streptococcus pneumoniae* TIGR4 340/341(99%) [0.0]	Periplasmic binding protein (58-308) [1.3e-29]
	orf3 (335)	FecCD transport system	WP_001244687.1/ *Streptococcus pneumoniae* TIGR4 333/335(99%) [0.0]	FecCD transport family (19-329) [1.7e-87]
	orf4 (335)	FecCD transport system	WP_001180357.1/ *Streptococcus pneumoniae* TIGR4 335/335(100%) [3e-177]	FecCD transport family (26-329) [1.3e-67]
	orf5 (264)	ABC transporter permease	WP_001180357.1/ *Streptococcus pneumoniae* TIGR4 263/264 (99%) [8e-161]	ABC transporter (19-167) [3.9e-32]
	orf8 (546)	Replication initiation protein		MobA/MobL family (18-245) [1.2e-81]
	orf10 (240)	DNA replication protein	WP_000224688.1/ *Streptococcus pneumoniae* TIGR4 23/242 (99%) [3e-174]	Phage replisome organizer, N terminal domain (9-125) [1.5e-27]
	tnp (448)	IS*Spn5*, family IS*1380*		
	orf15 (559)	DNA resolvase (recombinase)	WP_000031829.1/ *Streptococcus pneumoniae* TIGR4 550/559 (98%) [0.0]	Resolvase N terminal domain (7-157) [1.0e-34]; Recombinase (177-290) [6.9e-19]; Recombinase zinc beta ribbon domain (308-375) [2.1e-11]
	orf18 (252)	Carboxylate-amine/thiol ligases	WP_000262024.1/ *Streptococcus pneumoniae* TIGR4 249/252 (99%) [2e-167]	ATP-grasp domain, R2K clade family 3 (29-231) [5.6e-41]
	orf29 (284)	ADP-ribosylglycohydrolase	WP_000900015.1/ *Streptococcus pneumoniae* TIGR4 281/284 (99%) [0.0]	ADP-ribosylglycohydrolase (1-256) [2.4e-28]
	orf20 (294)	Diacylglycerol kinase	WP_000710115.1/ *Streptococcus pneumoniae* TIGR4 293/294 (100%) [0.0]	Diacylglycerol kinase catalytic domain (1-291) [8.76e-62]; YegS C-terminal NAD kinase beta sandwich-like domain (157-288) [1.4e-8]
	orf21 (579)	Alpha amylase	WP_015511339.1/ *Streptococcus pneumoniae* TIGR4 570/580 (98%) [0.0]	Alpha amylase, N-terminal ig-like domain (1-123) [5.1e-39]; Alpha amylase, catalytic domain (176-386) [9.1e-58]

Genomic Island	ORF (aa) [a]	Predicted Protein	Homologous Protein ID/Origin Identity (%) [E Value] [b]	Pfam Domain [c] (aa) [E Value]
GI-BM6001.3	*tnp* (198)	IS*Smi2*, family IS*1182*	WP_000276230.1/ *Streptococcus mitis* 73/558 (37%) [5e-33]	
	orf1 (64)	ROK family protein, truncated	AAK99988.1/ *Streptococcus pneumoniae* R6 61/64 (95%) [2e-36]	ROK family (3-59) [8.5e-8]
	orf2 (305)	Dihydrodipicolinate synthase family protein	AAK99989.1/ *Streptococcus pneumoniae* R6 301/305 (99%) [0.0]	DapA-like (7-293) [1.7e-58]
	orf3 (135)	Cytidine deaminase	AAK99991.1/ *Streptococcus pneumoniae* R6 135/135 (100%) [3e-88]	
	orf4 (162)	Phosphatidylglycerophosphatase A	AAK99992.1/ *Streptococcus pneumoniae* NCTC11902 162/162 (90.74%) [1e-93]	YutG/PgpA domain (42-155) [1.3e-20]
	orf5 (469)	Glycosyl hydrolase	AAK99993.1/ *Streptococcus pneumoniae* R6 469/469 (100%) [0.0]	Glycosyl hydrolase, five-bladed beta-propellor domain superfamily (20-203) [6.9e-06]
	orf6 (660)	ABC transporter ATP-binding protein	AAK99994.1/ *Streptococcus pneumoniae* R6 660/660 (100%) [0.0]	ABC-transporter-like, ATP-binding domain (26-189, 376-531) [2.1e-29, 7e-34]; Oligopepetide/dipeptide ABC transporter C-terminal domain (241-298, 583-618) [1.6e-15, 1.3e-05]
	orf7 (283)	ABC transporter permease	AAK99995.1/ *Streptococcus pneumoniae* R6 283/283 (100%) [2e-177]	ABC transporter type 1, transmembrane domain MetI-like (98-281) [1.9e-26]; Oligopeptide transport permease C-like, N-terminal domain (6-57) [4e-10]
	orf8 (316)	ABC transporter permease	AAK99996.1/ *Streptococcus pneumoniae* R6 316/316 (100%) [0.0]	ABC transporter type 1 GsiC-like N-terminal domain (1-103) [4.5e-10]; ABC transporter type 1 transmembrane domain MetI-like (111-316) [1.3e-42]
	orf9 (542)	ABC transporter substrate-binding protein	AAK99997.1/ *Streptococcus pneumoniae* R6 542/542 (100%) [0.0]	Solute-binding protein family 5 domain (84-454) [3.6e-64]
	orf10 (307)	Cyclically-permuted mutarotase family protein	AAK99998.1/ *Streptococcus pneumoniae* R6 307/307 (100%) [0.0]	Cyclically-permuted mutarotase family (14-304) [2.75e-17]
	orf11 (232)	N-acetylmannosamine-6-phosphate 2-epimerase	AAK99999.1/ *Streptococcus pneumoniae* R6 231/232 (99%) [1e-149]	Putative N-acetylmannosamine-6-phosphate epimerase (38-230) [1.5e-76]

Genomic Island	ORF (aa) [a]	Predicted Protein	Homologous Protein ID/Origin Identity (%) [E Value] [b]	Pfam Domain [c] (aa) [E Value]
GI-BM6001.4	*orf1* (408)	Tyrosine-type DNA integrase	CP102141.1/ *Streptococcus suis strain* DNR8 382/408 (83.09%) [0.0]	Phage integrase SAM-like domain (88-174) [2.0e-6]; Integrase, catalytic domain (213-372) [1.4e-20]
	orf2 (64)	Excisionase, putative		
	orf4 (469)	Cell division protein FtsK	CP102141.1/ *Streptococcus suis strain* DNR8 363/469 (76.12%) [0.0]	P-loop containing nucleoside triphosphate hydrolase (228-248) [1.8e-15]
GI-BM6001.5	*tnp* (81)	IS*6770*, family IS*30*, truncated	AAA68982.1/ *Enterococcus faecalis* 44/319 (24%) [5e-30]	
	orf1 (256)	Sulfite exporter	ACA35806.1/ *Streptococcus pneumoniae* Hungary19A-6 226/228 (99%) [7e-110]	Sulfite exporter TauE/SafE (7-247) [1.0e-31]
	orf2 (282)	Sulfatase	ACA36225.1/ *Streptococcus pneumoniae* Hungary19A-6 282/282 (100%) [0.0]	Sulfatase-modifying factor enzyme 1 (3-280) [1.1e-69]
	tnp (448)	IS*Spn5*, IS*1380* family	ACA36350.1/ *Streptococcus pneumoniae* Hungary19A-6 441/448 (98%) [0.0]	
	orf4 (451)	PTS system, IIC subunit	ACA37243.1/ *Streptococcus pneumoniae* Hungary19A-6 451/451 (100%) [0.0]	Phosphotransferase system, EIIC (29-380) [4.5e-51]
	orf5 (105)	PTS system, IIA subunit	ACA36919.1/ *Streptococcus pneumoniae* Hungary19A-6 104/105 (99%) [6e-50]	PTS system, Lactose/Cellobiose specific IIA subunit (8-100) [7.6e-37]
	orf6 (105)	PTS system, IIB subunit	ACA36309.1/ *Streptococcus pneumoniae* Hungary19A-6 105/105 (100%) [7e-53]	PTS system, Lactose/Cellobiose specific IIB subunit (6-98) [8.9e-23]
	orf7 (561)	PTS system		Carbohydrate binding domain (28-156) [3.2e-8]; Glycosyl hydrolases family 16 (219-444) [3.7-30]
	orf8 (298)	Sulfite exporter	ACA35833.1/ *Streptococcus pneumoniae* Hungary19A-6 294/298 (99%)	Sulfite exporter TauE/SafE (7-245) [1.0e-31]
	orf9 (420)	PTS system, IIC subunit	ACA37567.1/ *Streptococcus pneumoniae* Hungary19A-6 418/420 (99%)	Phosphotransferase system, EIIC (26-347) [3.1e-60]
	orf10 (392)	ROK family protein	ACA36800.1/ *Streptococcus pneumoniae* Hungary19A-6 389/392 (99%)	ROK family (81-353) [1.0e-32)]

[a] The number of amino acids of the predicted protein is shown in parenthesis. [b] Determined by compositional matrix adjustment. [c] Numbers in parentheses represent the part of the protein homologous to the Pfam domain.
